# Eleven-Month-Old Infants Preferentially Look at Helpers But Do Not Reliably Evaluate Inconsistent Actors

**DOI:** 10.1162/OPMI.a.370

**Published:** 2026-07-17

**Authors:** Brandon M. Woo, Adrian Tsang, J. Kiley Hamlin

**Affiliations:** Department of Psychological and Brain Sciences, University of California, Santa Barbara, Santa Barbara, CA, USA; Department of Psychology, University of British Columbia, Vancouver, BC, Canada

**Keywords:** cognitive development, social evaluation, impression formation, inconsistent behavior

## Abstract

Developmental psychology has provided some evidence that infants evaluate agents who engage in prosocial and antisocial acts: Infants selectively reach for and look at those who have treated third parties prosocially versus antisocially. This previous work has tended to present infants with actors whose behaviors are entirely prosocial or entirely antisocial. Studies introducing even minor variation into an actor’s behaviors have yielded null results with infants in the first year of life, suggesting that infants find it difficult to evaluate others who behave even mildly inconsistently. That said, it may be that past studies were poorly designed to test infants’ evaluations of inconsistent actors. The present, preregistered experiments further explored 11-month-old infants’ (*N* = 141) ability to evaluate inconsistent actors, using an online testing format and pre-recorded puppet shows depicting helping and hindering interactions. In Experiment 1, infants selectively looked to consistent helpers over consistent hinderers, conceptually replicating past findings via an online preferential looking procedure. We then adapted the same methods to examine infants’ evaluations of actors who behave mildly inconsistently. In Experiment 2, infants showed mixed evidence of evaluating inconsistent actors who performed a single inconsistent act after 4 consistent ones, preferring to look at more versus less prosocial actors in one preregistered analysis, but not in other analyses. Furthermore, infants did not find the inconsistency unexpected, suggesting successful evaluation may have stemmed from failing to notice the inconsistency and evaluating the inconsistent actor based on its earlier behavior. In Experiment 3, we put the single inconsistent action first, before the 4 consistent ones, to further test whether infants can evaluate inconsistent actors. Here, infants showed no evidence of successfully evaluating inconsistent actors. These findings suggest that while infants prefer agents whose actions are consistently prosocial versus antisocial, they struggle to evaluate those whose actions are even mildly inconsistent.

## INTRODUCTION

Imagine seeing a person offer their seat to someone else on the bus. This *behavior* was clearly kind, but is the *actor* a kind individual? Do they possess the trait of kindness? Are they likely to behave kindly in the future? Myriad social psychological theories are based on the idea that observers view others’ behaviors as indicative of their underlying, persistent characteristics (Cosmides & Tooby, 1992; Darwin, 2008; Heider, 2013; Henrich & Henrich, 2007; Jones & Davis, 1965; Joyce, 2007; Katz, 2000), guiding observers’ social decision-making and predictions of how individuals will behave in the future. This process is clearly complicated by the fact that people do not always behave consistently: Even deeply kind person are unlikely to behave prosocially in every situation, or even in the same situation across time; at best, overt behaviors are some noisy approximation of underlying traits. To generate accurate, holistic impressions of other people’s character, then, one must aggregate across multiple, potentially conflicting, behavioral data points. The present paper investigates the developmental origins of holistic impression formation, by assessing how preverbal infants respond to actors who are usually, but not always, prosocial or antisocial. Can infants evaluate inconsistent actors, and if so, how do they do it?

### Impression Formation in Adults

By observing other people’s behavior, adults quickly form character impressions: inferences concerning the kind of person other people *are* (e.g., whether they are “nice,” “mean,” “generous,” “greedy,” etc.). Adults generate these trait-like impressions even from sparse behavioral data (Ambady et al., 1995; Ambady & Rosenthal, 1992), even when they are not trying to (Uleman et al., 2006; Winter & Uleman, 1984), and sometimes even when there are good external explanations for an individual’s actions that should seem to preclude trait-like inferences (Brosch et al., 2013; Gilbert & Malone, 1995; Jones & Harris, 1967; Kubota et al., 2014; Ross, 1977; for cross-cultural differences, see Dean & Koenig, 2019).

Importantly, adults’ impressions flexibly incorporate information about the context in which trait-relevant behaviors occur. Indeed, the very same prosocial or antisocial act can lead to different attributions depending on context; for example, whereas apparently unprovoked harm might lead to the harmer being ascribed with traits like hot-headedness, aggression, or antisociality; harm viewed as deserved punishment might lead instead to the ascription of traits like trustworthiness and justice orientation (Barclay, 2006; Hamlin et al., 2011; Price et al., 2002). Flexible trait-attribution is readily observable in studies demonstrating that relationship contexts importantly impact impression formation; for example, an actor who restricts the freedoms of random strangers might be viewed as fundamentally bad, but a parent that restricts the freedoms of their child might be viewed as fundamentally good (see Aronson & Cope, 1968; Curry et al., 2019; Fiske, 1992; Fiske & Rai, 2014; Heider, 2013; Krasnow et al., 2016; Krems et al., 2023; Rai & Fiske, 2011; Tompkins & Powell, 2025). From this perspective, it would be unsurprising and even expected to encounter behavioral inconsistencies, so long as those inconsistencies can be interpreted in light of contextual information about deservingness and/or relationships.

Other times, though, an actor’s behavioral inconsistency cannot be explained by contextual information. How do people form impressions, given such unexplained inconsistencies? One straightforward strategy for inconsistency-informed impression formation would be to maintain a running mathematical average of each individual’s trait-relevant behaviors, and to generate (and adjust) one’s impressions based on the current state of that average. Yet, research has shown that adults often do not simply average across behaviors in this way and instead demonstrate various non-mutually exclusive biases in how inconsistency is incorporated into their impressions. One bias is typically referred to as a “primacy” or “first impression bias”; this reflects adults’ impressions prioritization of trait-relevant information acquired first. For example, when adults hear a target described with multiple, somewhat inconsistent, trait-related adjectives across time, their liking of the target is more impacted by those adjectives they heard earlier versus later (Anderson, 1965; see also, Anderson & Hubert, 1963; Kim et al., 2020, 2022). A second common bias is known as a “negativity bias”; this refers to adults’ impressions tending to weigh information about antisocial behaviors more strongly than information about prosocial ones. For example, when a person is described with both a negative adjective (e.g., dishonest) and a positive one (e.g., kind), the negative adjective more greatly influences how much adults like the person (Skowronski & Carlston, 1989; see also, Baumeister et al., 2001; Ferguson et al., 2019; Kanouse & Hanson, 1987; Rozin & Royzman, 2001). Such impression-related biases reveal that the social computations underlying impression formation are not well described by simple averaging processes.

### Impression Formation in Development

How and why do capacities for impression formation, alongside biases suggestive of difficulty with or resistance to engaging in basic averaging in cases of inconsistency, emerge in human development? At least two possibilities exist. The first is that adults engage in rapid and sometimes biased impression formation as the result of a lifetime of experience with observing and making sense of others’ behaviors across time. For instance, adults may have learned that individuals tend to behave largely consistently over time, and therefore that forming impressions based on early-observed behaviors is a useful shortcut. Relatedly, adults may have learned that those times they failed to prioritize antisocial behaviors in their impressions were especially costly, leading them to weight antisocial behaviors—even rare ones—relatively strongly.

On the other hand, impression formation may not (solely) reflect learning within individual lifespans, but also over evolutionary time (see Rozin & Royzman, 2001; Schaller, 2008). To the extent that generating impressions based on observed behaviors was useful for survival, abilities to do it may have been selected for; those impressions that happened to be weighted toward early-encountered and/or negative behaviors may have been particularly survival-relevant. If so, abilities to generate impressions, perhaps including the various biases known to impact them, might emerge as early as infancy, prior to extensive opportunity for social learning (see Hamlin, 2013b; Woo & Hamlin, 2022; Woo et al., 2022). Crucially, these abilities would be reinforced and/or shifted by social experiences amassed throughout the lifespan, but they would not be wholly generated from them. Do preverbal infants use their observations of others’ behavior to generate impressions? If so, are their impressions subject to the same biases adults’ are?

### Do Infants Generate Social Impressions?

**Figure 1.**
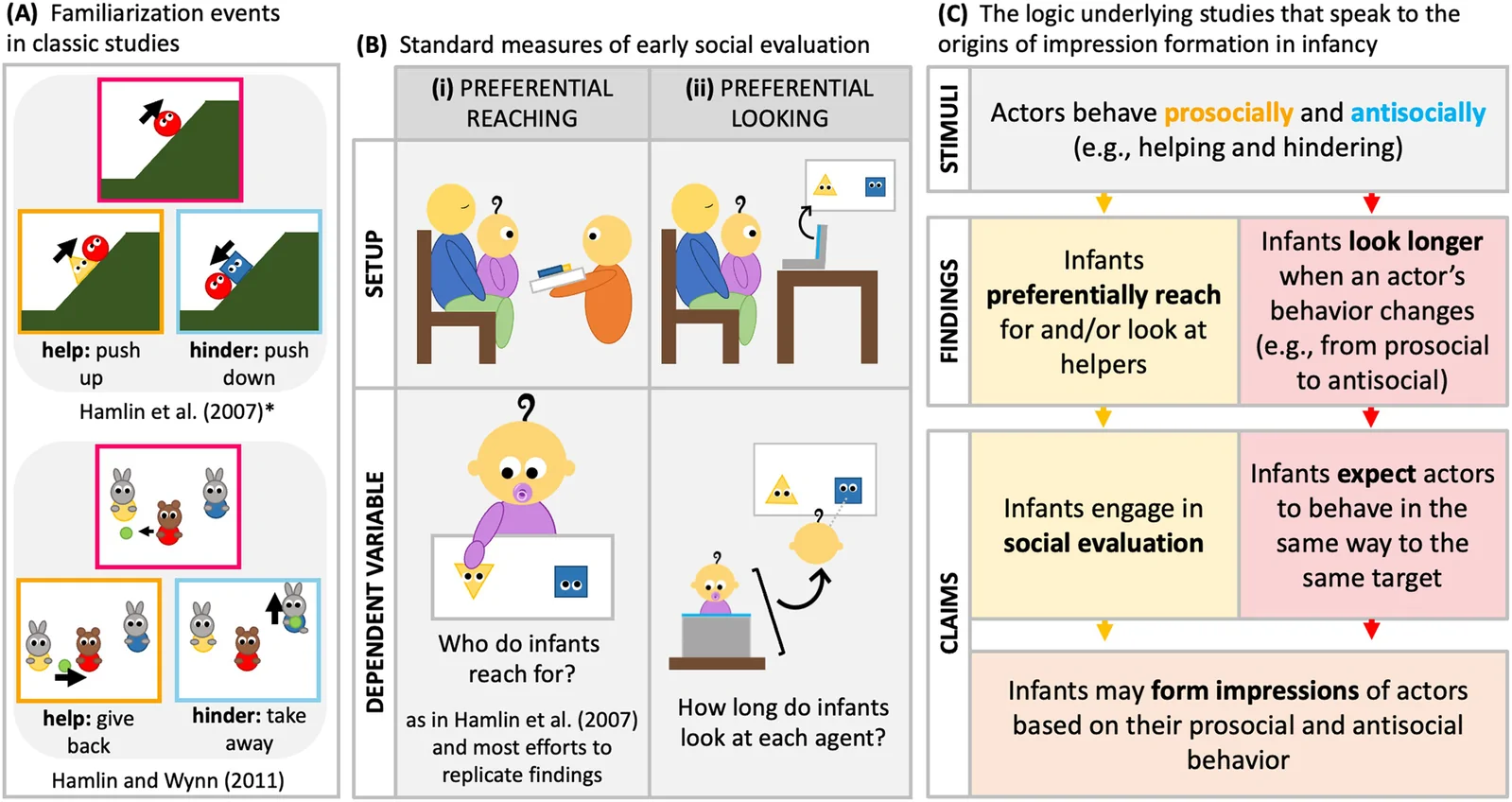
Examples of familiarization events in classic studies probing infants’ evaluation of helping and hindering (A), common measures of infants’ evaluations (B), and the logic underlying studies that speak to the origins of impression formation in infancy (C). Findings that infants engage in social evaluation and expect actors to behave in the same way, at least to the same target and sometimes to novel targets, provide evidence that infants may form impressions of actors based on their prosocial and antisocial behavior.

A large body of evidence suggests that infants distinguish a variety of prosocial from antisocial acts and actors, positively evaluate prosocial actors, and negatively evaluate antisocial ones (for review, see Woo et al., 2022). For example, in one study 3- and 5-month-old infants watched displays in which a puppet protagonist played with, and then dropped, a ball. During alternating events, a helper puppet always returned the ball, while a hinderer puppet always took the ball away (Figure 1). Following habituation (e.g., repeated exposure until a pre-set reduction in attention was reached) to these events, infants were presented with the helper and hinderer side-by-side, by an experimenter unaware of who had done what. Five-month-old infants preferentially reached toward the helper over the hinderer, and 3-month-olds preferentially looked at the helper (Hamlin & Wynn, 2011). These looking and reaching preferences have been taken as evidence that infants engage in social evaluation. To date, this and many related papers have provided evidence that infants evaluate those who help versus hinder a variety of unfulfilled goals, including climbing a steep hill (Hamlin, 2014; Hamlin et al., 2007; Tan & Hamlin, 2022; Woo, Liu, et al., 2024), opening a box containing an object (Hamlin & Wynn, 2011; Steckler et al., 2017; Woo & Spelke, 2023a, 2023b), collecting a toy off a high shelf (Van de Vondervoort et al., 2018; Woo et al., 2017), and accessing a preferred toy behind a barrier (Hamlin, Ullman, et al., 2013).

In addition to helping and hindering, studies have demonstrated that infants prefer prosocial to antisocial others in a range of social contexts; infants prefer actors who comfort others over actors who ignore others in distress (Biro, 2023), actors who protect others over actors who cause harm (Kanakogi et al., 2017), and actors who distribute resources fairly over actors who distribute resources unfairly (Geraci & Surian, 2011; Lucca et al., 2018; for review, see Woo et al., 2022). Across studies, researchers have used a wide variety of agents (humans, puppets, animated shapes), ways of depicting prosocial and antisocial acts (live versus video), and ways of assessing infants’ evaluations (preferential looking, reaching, giving, taking, praise, admonishment; see Woo et al., 2025), suggestive that infants’ evaluations are unlikely to stem from idiosyncratic, non-social features of any individual study. Although the broader literature includes several failures to replicate evidence that infants preferentially reach for helpers over hinderers (Cowell & Decety, 2015; Nighbor et al., 2017; Salvadori et al., 2015; Schlingloff et al., 2020), including a large-scale replication attempt involving dozens of labs and hundreds of infants (Lucca et al., 2025), current meta-analytic estimates including those failures suggest that infants’ overall preference for prosocial others is robust (Margoni & Surian, 2018; see also, Margoni & Tan, 2026).

Some have proposed that infants’ social evaluations may reflect early precursors to impression formation, supporting infants’ social decision-making and predictions of others’ future behavior (Woo & Hamlin, 2022; Wynn, 2009). Although tendencies to look at or reach for one actor over another could of course reflect multiple underlying processes, claims that infants form impressions are bolstered in part by studies suggesting that infants expect individuals to behave in valence-consistent ways (e.g., prosocially or antisocially) over time towards the same target (Pepe et al., 2026; Shimizu et al., 2018; see also, Taborda-Osorio et al., 2019; Tatone et al., 2015) and, in multiple studies, towards new targets (Gill & Sommerville, 2023; Surian et al., 2018; Ting & Baillargeon, 2021; Zeng et al., 2026; see also, Pepe et al., 2026; Thomas, 2026) (Figure 1C). Whereas the evidence that infants form expectations of behavior towards the same target could suggest that they are forming inferences about relationships (Thomas, 2026), the evidence that infants form expectations of behavior towards new targets suggests that they may infer traits. In many of these studies, infants demonstrated expectations of consistency, even when the physical characteristics of the actions changed across time. These findings suggest that infants’ expectations are unlikely to be explained by patterns of low-level association. Infants’ expectations and evaluations may instead be consistent with the possibility that infants (like adults) attribute some underlying mental states, dispositions, or traits to prosocial and antisocial actors. If so, these capacities could serve as a foundation for adult impression formation, guiding social decision-making (Figure 1).

### Updating Impressions in Infancy

Despite evidence that infants seem to *notice* trait-inconsistent actions, research on early social *evaluation* has almost exclusively presented infants with consistent actors who act consistently, either entirely prosocially or entirely antisocially. But infants’ everyday lives likely include actors whose behaviors are at least somewhat inconsistent. Do infants evaluate these inconsistent individuals, and if so, how? Evaluating inconsistent actors is presumably more challenging than evaluating consistent actors, as it involves making sense of different and potentially conflicting behaviors. Thus, studying infants’ evaluations of inconsistent actors could shed light on the depth, limits, and basis of early social evaluation.

**Figure 2.**
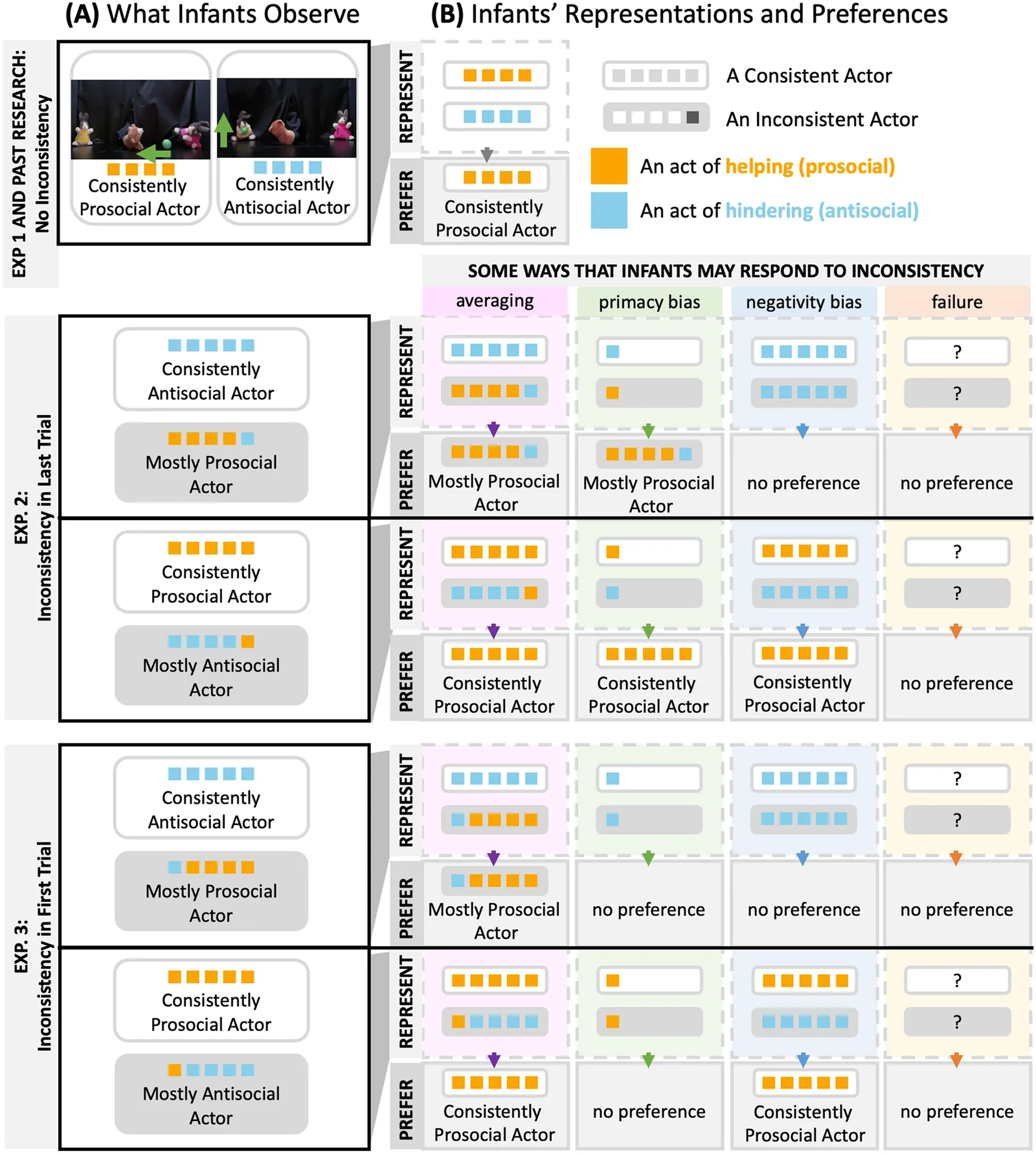
A conceptual schematic for what infants have observed in past research and in the current Experiments 1, 2, and 3 (A), the various representations that infants might have of those actors (B), and the preferences that infants might have based on those representations (B). Across panels, orange squares indicate acts of helping, blue squares indicate acts of hindering, white curved rectangles indicate an actor who behaved consistently, and gray curved rectangles indicate an actor who behaved inconsistently.

There are at least four possibilities for how infants may respond to inconsistent actors (see Figure 2). First, infants may engage in a simple averaging process as described above, maintaining and adjusting an unbiased summary of each individual’s positive and negative behaviors and preferring actors who are comparatively more prosocial overall. Second, infants could weigh initial behaviors relatively strongly, in line with a primacy bias. If so, then they should prefer actors who were initially prosocial over actors who were initially antisocial, irrespective of later behaviors. Third, infants may process inconsistencies differently depending on their valence (prosocial versus antisocial); for instance, placing more weight on rare negative information than on rare positive information (i.e., a negativity bias). If infants evaluate actors in line with a negativity bias, then they should prefer a consistently prosocial actor over a mostly antisocial one, but not as strongly distinguish a consistently antisocial actor and a mostly prosocial actor, given that each has behaved antisocially some of the time. Finally, inconsistency could simply overwhelm infants’ limited capacities for social evaluation, leading them to fail any time they encounter any actor behaving inconsistently.

To date, we are aware of only two papers that have examined infants’ evaluations of inconsistent actors (see Shimizu et al., 2018; Steckler et al., 2017). In both of these papers, when younger, preverbal infants (9-month-olds in Steckler et al., and 6-, 9-, and 12-month-olds in Shimizu et al.) were presented with inconsistent actors who had behaved both prosocially and antisocially, infants did not show evidence of reliably evaluating them (that is, by reaching for the actor who was relatively more prosocial). In contrast, older infants (e.g., 15- to 18-month-olds, studied by Shimizu et al.) preferred reaching for the relatively more prosocial actor. Together, these papers suggest that infants may have difficulty evaluating individuals who behave even a little inconsistently, but that they may be increasingly able to do so with age.

Alternatively, perhaps young infants actually *can* update impressions to evaluate inconsistent actors, but methodological issues of past work prevented them from doing so. At least two methodological issues seem relevant. First, the prosocial and antisocial actions used to establish infants’ impressions in past work may not have been ideal. Indeed, both Shimizu et al. (2018) and Steckler et al. (2017) primarily utilized the “box scenario” in which a protagonist is helped and hindered in its goal to open a box (Hamlin & Wynn, 2011); a meta-analysis on infants’ social evaluations (Margoni & Surian, 2018) suggested that infants’ tendency to prefer helpers over hinderers is stronger in other scenarios. Perhaps infants could successfully form impressions of inconsistently prosocial and antisocial actors if a different helper/hinderer scenario were used. Second, at least in Steckler et al. (2017), infants may not have been given sufficient evidence of an inconsistent actor’s *usual* behavior prior to encountering the inconsistency. Infants were shown the inconsistent actor acting only twice in one way before being shown the opposite; this small amount of initial evidence may have rendered their initial impression relatively weak, particularly if box-scenario displays are difficult to understand. It may be that weakly positive versus negative impressions are sufficient for infants to distinguish prosocial from antisocial others where no inconsistencies exist (they have seemed to be in past work; Hamlin et al., 2011), but nevertheless insufficient once even minor inconsistencies are introduced.

### The Present Experiments

The current studies were designed to further investigate infants’ ability to evaluate inconsistent actors, while overcoming the possible explanations for infants’ failures in past work. We conducted three experiments with 11-month-old infants. All experiments used the “ball scenario” from Hamlin and Wynn (2011), in which a protagonist loses its ball and other actors either return the ball or take it away; this scenario was chosen because a meta-analysis of infants’ and toddlers’ social evaluations (2018) suggests it has a larger effect size than the box scenario (see also, Margoni & Tan, 2026; Tan, 2024). In Experiment 1, we first attempted to conceptually replicate the finding that infants prefer looking at helpers over hinderers in the ball scenario, when no one acts inconsistently. Replicating this finding was important, both due to the replication failures in the literature described above and because infants were tested online via Zoom, where infants’ social preferences were measured through preferential looking rather than reaching and where no experimenter presented the puppets. Following successful replication, Experiment 2 tested infants’ evaluation of inconsistently prosocial or antisocial actors, by depicting inconsistent actors acting the same way 4 times in a row before (for inconsistent actors) inconsistency was introduced during the last trial. We included two additional familiarization events, relative to past work (Steckler et al., 2017), to more clearly establish how all actors usually behave before changing anything; notably, this design also allowed us to assess whether or not infants looked longer following the behavioral change. Despite looking time analysis not having been the primary motivation underlying our design, it would nevertheless allow us to assess whether or not infants showed any evidence of expecting actors to behave consistently. Finally, Experiment 3 examined whether the timing of the inconsistency mattered, by putting the inconsistent act first rather than last.

Overall, we reasoned that if infants evaluations are based on a running average of actors’ prosocial and antisocial behavior across time, then they should preferentially look at the relatively more prosocial actor across all experiments. In contrast, distinct patterns of response between experiments could indicate a reliance on (i) one or more of the “impression biases” that adults are subject to, (ii) other heretofore unspecified impression biases, or (iii) a total inability of early social evaluation to incorporate inconsistency at all (Figure 2).

## EXPERIMENT 1: EVALUATIONS OF CONSISTENT ACTORS (REPLICATION OF PAST RESEARCH)

We first sought to attempt to replicate the finding that, when individuals behave consistently, infants prefer actors who help over actors who hinder. We assessed infants’ evaluations using preferential looking, in which infants’ evaluations of two actors is inferred from their relative looking to each over a set time period, following other research using this measure to probe infants’ and toddlers’ social evaluations (e.g., Geraci et al., 2022; Geraci & Surian, 2023; Hamlin & Wynn, 2011; Hamlin et al., 2010; Powell & Spelke, 2018; Woo, Liu, et al., 2024; Woo & Spelke, 2023a, 2023b). To our knowledge, infants’ evaluations of helpers over hinderers in the ball scenario had not been previously demonstrated online, and so it was important for us to examine it prior to addressing our main research question.

### Methods

Hypotheses, methods, and analyses for the present experiments were preregistered on the Open Science Framework. The stimuli, data, and code are hosted at https://osf.io/879ys/, and the preregistration for the present experiment is hosted at https://osf.io/n7qmp/.

#### Participants.

Twenty-four 11-month-olds (8 girls; mean age = 10 months, 28 days, range = 10;16–11;17) participated. An additional eight infants began the experiment but were not included in the final sample due to technical issues (*n* = 4), fussiness (*n* = 2), procedural error (*n* = 1), and inattentiveness (*n* = 1). Parent or guardian consent was obtained prior to participation.

##### Sample Size Justification.

We used both pilot data (collected with the present methods) and previously published data (Hamlin & Wynn, 2011) for power analyses to determine an appropriate sample size. In pilot data (*n* = 8), we examined the amount of time that infants looked at the helper over the hinderer. On average, infants looked to the helper for 12.94 s and to the hinderer for 10.02 s. We ran a power analysis to determine what the sample size should be to detect a looking preference of the same effect size with sufficient power in the mixed-effects model detailed above. With 24 infants, we would have 86% power to detect a looking preference of the same effect size.

Additionally, we ran power analyses based on published data by Hamlin and Wynn (2011) using the present method, but in a lab setting. There, 10/12 infants preferred the helper over the hinderer. We found that with 24 infants, we would have 94% power to detect a reaching preference of the same effect size. Although we are not assessing reaching preferences here, we assumed that looking and reaching preferences should be consistent with each other and should both reflect infants’ evaluations (see, e.g., Hamlin et al., 2010). We therefore decided to test 24 infants.

#### Procedures.

Infants viewed events from their parent’s lap or from a highchair, from their own homes. We followed best practices for online testing recommended by Chuey et al. (2021). Parents were instructed to sit quietly, look away, not attempt to influence their infants’ attention, and minimize distractions in the room (e.g., objects near the computer). The experimenter displayed the events on the parent’s device (e.g., a laptop) over a video call, using YouTube’s playlist feature (on an ad-free version of YouTube), while sharing screens with the parent. The experimenter worked with parents to ensure that they could not see themselves (i.e., self-view was turned off), that the only visual on the participants’ screens was the study stimuli, and that audio was audible. The experimenter used a physical opaque folder to hide the part of their screen that displayed the stimuli that the infants viewed, so that the experimenter remained unaware of any counterbalanced variables (e.g., the identity of each actor; see counterbalancing information below).

##### Helping and Hindering Familiarization Events (as in Hamlin & Wynn, 2011).

All events began identically. A curtain rose to reveal a green ball at the center of a stage. Two gray rabbits, wearing different shirts (pink versus yellow), sat at the stage’s corners. A protagonist (a brown bear) picked up the ball. It jumped twice, and on a third jump it dropped and retrieved the ball; it repeated this sequence three times. When the protagonist jumped after the third sequence, the ball fell toward one side and the rabbit there intervened. During helping events, the rabbit grabbed the ball. The protagonist turned to the rabbit, opening its arms as though to ask for the ball back. The rabbit turned toward the protagonist, and the two puppets faced forward. The protagonist then turned and opened its arms again; the rabbit turned, and both faced forward again. On the protagonist’s third turn, the rabbit rolled the ball toward the protagonist, who caught it. The rabbit ran off stage, and the protagonist faced forward, holding the ball. Hindering events were like helping events, except that on the protagonist’s third turn, the rabbit ran off-stage with the ball. The protagonist then faced forward, empty-handed.

**Figure 3.**
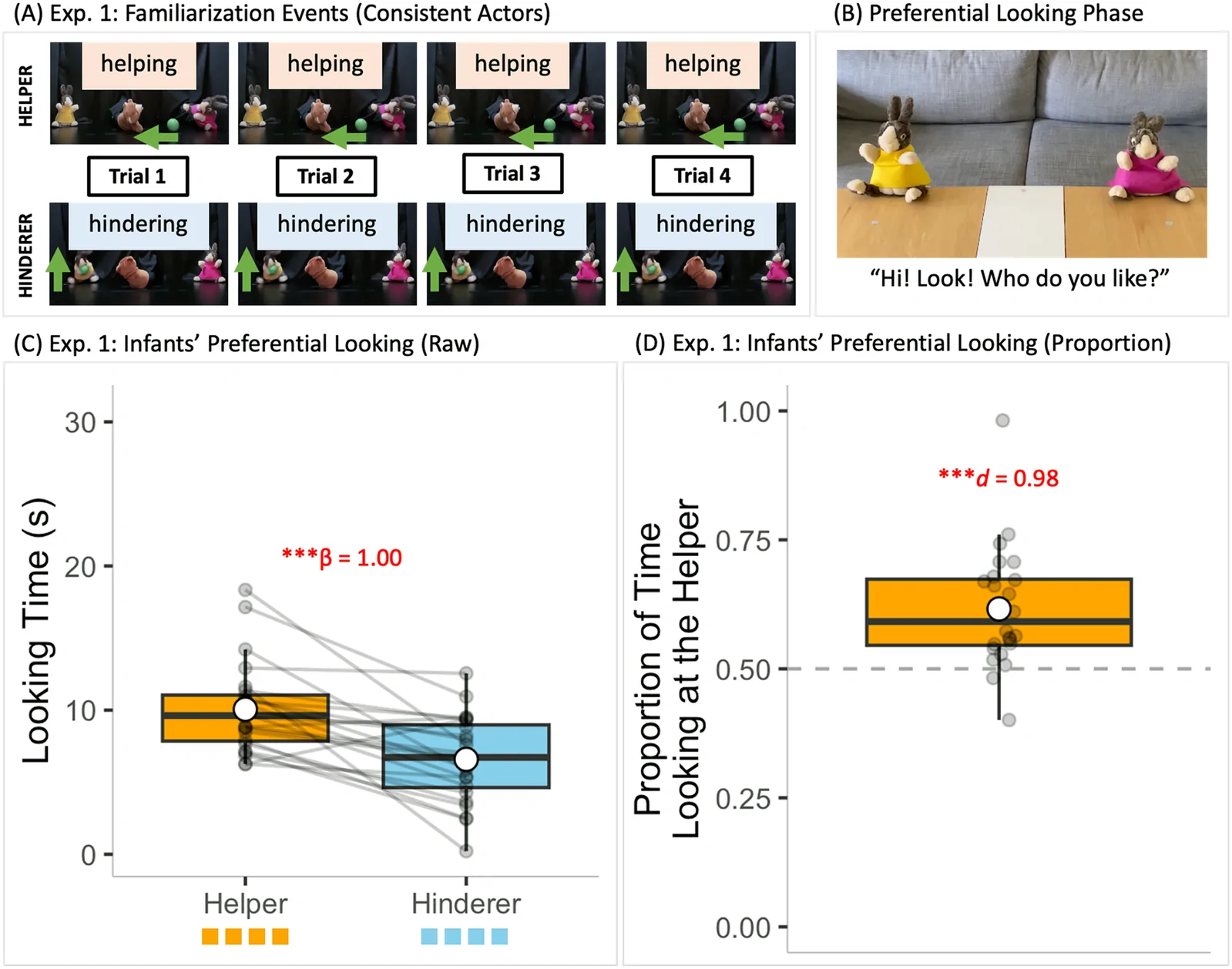
Experiment 1’s familiarization events (A), a screenshot of the preferential looking phase (B), and infants’ looking during the preferential looking phase as raw data (C) and in terms of the proportion of time looking at the Helper (D). For panel C, the colored boxes below the box plots indicate the behaviors that an actor engaged in (helping in orange, hindering in blue) across its four trials. White circles indicate means, horizontal lines within boxes indicate medians, pairs of connected dots in C indicate data from a single child, and boxes indicate interquartile ranges. The beta coefficient (*β*) and Cohen’s *d* are measures of standardized effect size (***p < .001, two-tailed).

During all events, action paused once the acting rabbit was off-stage. Infants’ looking was recorded from this point by the experimenter who was blind to condition using the coding program jHab (Casstevens, 2007), continuing until infants looked away for 2 consecutive seconds or 30 seconds elapsed. Infants saw a total of 8 trials, with 4 trials from each actor in alternation (Figure 3A). One actor, the Helper, always helped the bear by returning the ball, and the other actor, the Hinderer, always took the ball away. A second coder recoded looking during the familiarization events of 25% of participants from Experiments 1–3, and we found that the intraclass correlation coefficient for the two coders’ times was 96% (95% CI [0.95, 0.97]).

##### Preferential Looking Phase.

Following the trials, an experimenter presented the infants with a video of the puppets (Figure 3B). The two rabbits appeared on opposite sides of the screen and a prerecorded voice played saying “Hi! Look! Who do you like?” three times, once every 10 s, over a 30 s period, as the rabbits’ hands moved as though they were clapping. A live coder coded all looking at the actors during this period. Infants’ looking during the studies was video recorded, and to assess reliability, a second coder recoded the video recordings of 25% of participants from Experiments 1 to 3. We found that the intraclass correlation coefficient for the two coders’ looking times was 93% (95% CI [0.89, 0.95]).

##### Counterbalancing.

We counterbalanced: (i) the color of the Helper (pink/yellow), (ii) the side of the Helper across the events and the preferential looking phase, and (iii) the order of the Helper (first/second). This resulted in eight possible versions of the counterbalanced stimuli; we randomly assigned each participant to one version. For all of the present experiments, the research team relabeled these versions and shuffled them, so that the specific combination of counterbalanced variables was unknown to experimenters when they randomly chose to run a particular version.

### Results

We analyzed the data in three ways. If there is strong evidence that infants preferred one actor over the other, then the results of the three analytic approaches should converge. First, we ran a preregistered mixed-effects model on the data in the preferential looking phase. In this model, looking time was the dependent variable, and the actor type (Helper versus Hinderer) was the fixed effect, with subject ID as a random effect. We found that infants looked longer in the preferential looking phase to the Helper (mean = 10.06 s, *SD* = 3.12 s) than to the Hinderer (mean = 6.58 s, *SD* = 2.97 s); *β* = 1.00, 95% CI of *β* [0.61, 1.39], *b* = 3.47, *t*(24) = 5.15, *p* < .001).

Second, after collecting data and running the mixed-effects models in Experiments 1–3, we realized that mixed-effects models did not account for one feature of our design: that both actors were present on screen at once during the preferential looking phase, and so infants’ attention to one agent necessarily impacted their looking to the other agent (that is, reduced it). We note that analyses of the raw preferential looking time data are standard in the study of early social evaluation (e.g., Geraci et al., 2022; Hamlin & Wynn, 2011; Hamlin et al., 2010; Powell & Spelke, 2018; Woo, Liu, et al., 2024; Woo & Spelke, 2023a, 2023b). Even so, we amended our preregistration to include an additional analysis, for which we calculated the proportion of the time that each infant looked at the Helper out of their total looking to either of the two actors and ran a one-sample *t*-test against chance (0.50). Consistent with our mixed-effects models, here infants looked at the Helper more than would be expected by chance (proportion_helper_ = 0.61, 95% CI of the proportion [0.56, 0.66]), *t*(23) = 4.82, *p* < .001; Cohen’s *d* = 0.98; Figure 3D).

Finally, in an exploratory analysis we categorized individual infants as having looked more at either the Helper or the Hinderer during the preferential looking trial. Twenty-two of 24 infants looked more at the Helper (binomial *p* < .001; Cohen’s *g* = 0.41).

### Discussion

Experiment 1 replicated the finding that infants prefer individuals who help others over individuals who hinder others in the ball scenario, using an online format and preferential looking. We next moved on to exploring our key research question: whether and how infants evaluate inconsistent actors.

## EXPERIMENT 2: EVALUATIONS OF INCONSISTENT ACTORS; INCONSISTENCY LAST

Whereas Experiment 1 contrasted two consistent actors (one who always helped and one who always hindered), Experiment 2 contrasted a consistent actor with a mildly inconsistent one. Specifically, Experiment 2’s inconsistent actors either (i) helped a protagonist (giving its ball back) 4 times before hindering the protagonist (taking its ball away) once (the Mostly Prosocial Inconsistent Actor Condition) or (ii) hindered the protagonist 4 times before helping the protagonist once (the Mostly Antisocial Inconsistent Actor Condition). As in Steckler et al. (2017), the consistent actor always acted consistently and maximally different from the inconsistent actor; that is, always hindering in the Mostly Prosocial Inconsistent Actor Condition and always helping in the Mostly Antisocial Inconsistent Actor Condition. Thus, our design intentionally minimized inconsistency to give infants the best possible chance of successfully distinguishing the two characters.

Given evidence that infants can evaluate actors on the basis of a single prosocial or antisocial act (Hamlin, 2014; Hamlin et al., 2011), we reasoned that changing an actor’s behavior during the actor’s last familiarization event would be sufficient for infants to recognize that an actor had behaved inconsistently. To assess this more formally, we examined infants’ attention during the last familiarization event for each actor; one was consistent and the other was inconsistent with each actor’s previous behavior. We anticipated that infants may look longer when the inconsistent actor behaved inconsistently, relative to when the consistent actor behaved consistently.

Following familiarization, infants were presented with the consistent and inconsistent actors and their attention to each was measured over 30 seconds. If 11-month-olds use something like an averaging process to evaluate the inconsistent actors, then they should preferentially look at the actor who helped relatively more versus less, irrespective of consistency and valence (see Figure 2). Specifically, when the consistent actor always helps and the inconsistent actor mostly hinders (i.e., is a Mostly Antisocial Actor), infants should prefer the consistent actor. When the consistent actor always hinders and the inconsistent actor mostly helps (i.e., is a Mostly Prosocial Actor), infants should preferentially look at the Mostly Prosocial Actor. Alternatively, infants’ evaluations may reflect some bias (e.g., a negativity bias; Figure 2), following the adult literature. Finally, infants may not demonstrate any clear preferences in their looking behavior; this would suggest that adjustments made relative to Steckler et al. (2017) were insufficient to allow infants to evaluate inconsistent actors.

### Methods

The preregistrations for Experiments 2 and 3 are hosted at https://osf.io/bjy5x/.

#### Participants.

Forty-eight 11-month-olds (21 girls; mean age = 10 months, 26 days; range = 10;14–11;26) participated. Infants were randomly assigned to the “Mostly Antisocial Inconsistent Actor” Condition, wherein the inconsistent actor hindered 4 times and helped once, or the “Mostly Prosocial Inconsistent Actor” Condition, wherein the inconsistent actor helped 4 times and hindered once (*n* = 24 per condition). An additional four infants began the experiment but were not included in the final sample due to technical issues (*n* = 2), fussiness (*n* = 1), and inattentiveness (*n* = 1).

##### Sample Size Justification.

We used pilot data (collected with the methods for Experiment 2) for power analyses to determine an appropriate sample size. In pilot data (*n* = 9), we examined the amount of time that infants looked at the more prosocial over the more antisocial actor by condition. When the inconsistent actor was usually antisocial (the Mostly Antisocial Inconsistent Actor Condition), infants looked at the Mostly Antisocial Actor for 8.53 s and the Consistently Prosocial Actor for 9.75 s. When the inconsistent actor was usually prosocial (the Mostly Prosocial Inconsistent Actor Condition), infants looked at the Consistently Antisocial Actor for 6.65 s and the Mostly Prosocial Actor for 13.14 s. Although this effect appeared qualitatively larger when the inconsistent actor was usually prosocial, there was a very small amount of data per condition.

Nonetheless, we ran a power analysis to determine what the sample size should be to detect a looking preference between the consistent and inconsistent actors of the same effect size with sufficient power as in the mixed-effects model detailed above. Given the qualitative trend above, we first examined the sample size necessary to detect an interaction the size of that in the pilot data (*β* = 1.09). We found that, with 48 infants (24 per condition), we would have 100% power (within machine precision) to detect this interaction. We also ran another power analysis, but without the inconsistent actor’s usual act in the model, to detect the main effect of looking to the more prosocial actor (*β* = 0.62). With 48 infants (24 per condition), we would have 99% power to detect a looking preference for the more prosocial actor. Thus, our overall analysis was sufficiently powered irrespective of whether the interaction between conditions observed during piloting was stable. We note that power was relatively high here, and this likely reflects that infants’ preferential looking revealed large effect sizes in the pilot data.

In the pilot data, we found almost no evidence that infants looked longer when the inconsistent actor’s behavior changed. Overall, infants looked 13.30 s at the 4th trial, after the inconsistent actor behaved as it had in the earlier trials. In the 5th trial (the one containing the inconsistency), they looked 9.64 s. Given that infants appeared to not look longer following this change, the uncertainty of online testing methods, and that our studies were designed to assess preference rather than expectations per se, we chose to focus our power analyses on infants’ preferential looking. That said, we still planned to examine infants’ expectations of the actors’ behaviors in the final sample.

#### Procedures.

The procedure was like that of Experiment 1, except that there was an additional, fifth trial for each actor. Thus, infants saw a total of 10 trials. We chose 5 trials per actor so that there would be the same evidence of consistent behavior (4 trials) prior to any inconsistency as they had seen in Experiment 1, given that Experiment 1 suggested that 4 trials was sufficient for infants to generate evaluations (see Figure 3). Infants were randomly assigned to the “Mostly Antisocial Inconsistent Actor” Condition, in which the inconsistent actor hindered 4 times and helped once, and the consistent actor helped 5 times; or the “Mostly Prosocial Inconsistent Actor” Condition, in which the inconsistent actor helped 4 times and hindered once, and the consistent actor hindered 5 times.

**Figure 4.**
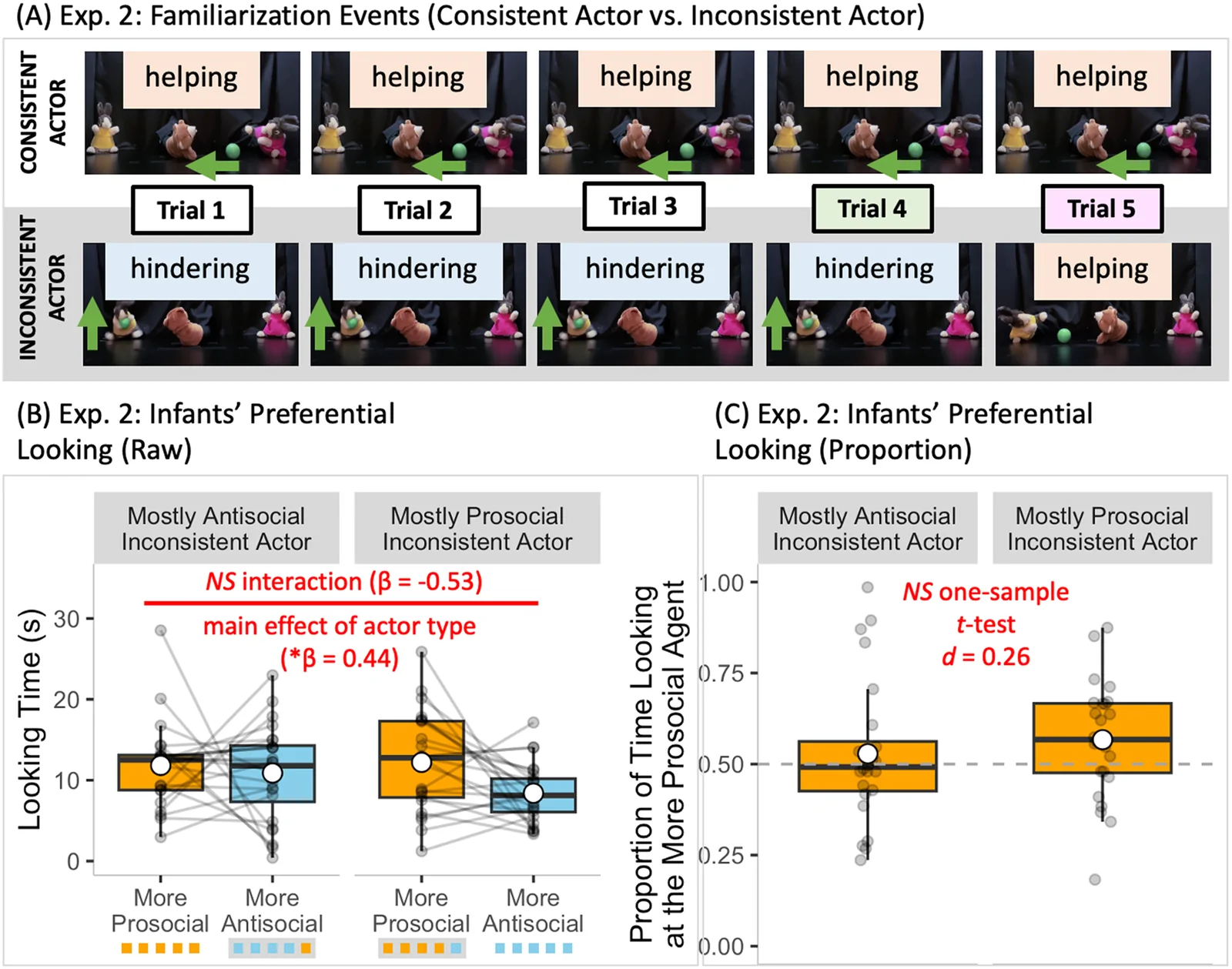
Experiment 2’s familiarization events (A) and infants’ preferential looking as raw data (B) and in terms of the proportion of time looking at the more prosocial actor (C). Orange indicates helping or prosocial behavior, blue indicates hindering or antisocial behavior, green indicates the fourth trial for an actor (consistent for both actors), and purple indicates the fifth trial for an actor (inconsistent only for the inconsistent actor). The screenshots in panel A are representative of Experiment 2’s “Mostly Antisocial Inconsistent Actor” Condition; the “Mostly Prosocial Inconsistent Actor” Condition is not shown. Following the familiarization events, we assessed infants’ evaluations with a preferential looking phase (as in Experiment 1; see Figure 2B). In panel B, the colored boxes below box plots indicate the behaviors that an actor engaged in (helping in orange, hindering in blue) across its five trials; and the colored boxes in gray indicate that that actor was inconsistent. In the box plots, white circles indicate means, horizontal lines within boxes indicate medians, pairs of connected dots indicate data from a single child, and boxes indicate interquartile ranges. The beta coefficients (*β*) and Cohen’s *d* are measures of standardized effect size (*NS* *p* > .05; *p < .05, two-tailed).

The consistent actors performed the same action in all 5 of their trials (either always returning the ball, or always taking the ball away), whereas the inconsistent actors acted opposite to their respective consistent actor for their first 4 trials, and then switched their behavior to behave similarly to the consistent actor on the last trial (Figure 4). To reduce working memory demands, each actor completed its trials consecutively (instead of on alternating trials), as in Steckler et al. (2017). That is, one actor acted in all 5 of its trials before the other began acting. Steckler et al. (2017) argued that, when actors’ behaviors change, it may be easier to update an impression when each actor acts in succession, rather than in alternating order; we therefore had each actor complete its trials consecutively in the present experiment.

Coding was identical to that of Experiment 1.

##### Counterbalancing.

We counterbalanced: (1) the color of the consistent actor (pink/yellow); (2) the side of the consistent actor across the events and the preferential looking phase (left/right); and (3) the order of the consistent actor (first/second).

### Results

#### Expectations of Consistency.

We asked whether infants formed expectations for consistency, as well as whether expectations varied by valence. Because looking time data during familiarization events are often positively skewed (Csibra et al., 2016), we preregistered that we would first fit both a gamma distribution and a normal distribution to the data, and then based on which was a better fit, run either a gamma mixed-effects model or a standard one. We found that a gamma distribution fit the data better than did a normal distribution; we therefore ran a gamma mixed-effects model. Looking time was the dependent variable, with actor type (Consistent/Inconsistent Actor; within-subjects), trial order (corresponding with the trials involving the switch in behavior for inconsistent actors and the one before that; within-subjects), the inconsistent actor’s usual act (Helping/Hindering; between-subjects), and all possible interactions as fixed effects. Participant ID was a random effect. The fixed effects of actor type, order, and usual act were centered.

**Figure 5.**
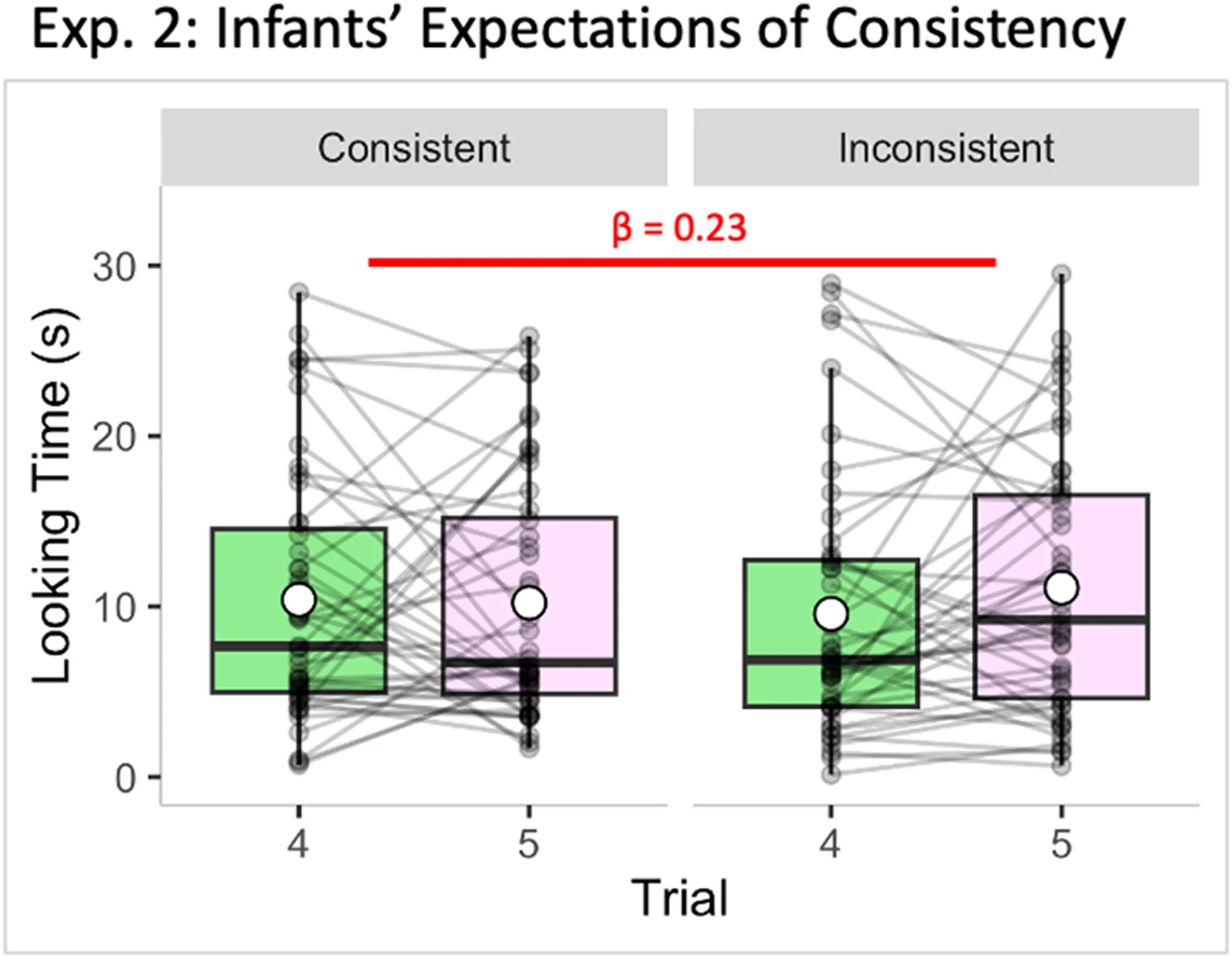
Infants’ expectations in Experiment 2. Green indicates the fourth trial for an actor (consistent for both actors), and purple indicates the fifth trial for an actor (inconsistent only for the inconsistent actor). White circles indicate means, horizontal lines within boxes indicate medians, pairs of connected dots indicate data from a single child, and boxes indicate interquartile ranges. The data are collapsed across actor conditions. The beta coefficient (*β*) is a standardized measure of effect size.

Analyses revealed no significant interactions (two-way or three-way) or main effects (all *p*s > .18). For both conditions, the change in infants’ looking between the fourth and fifth trials was not significantly different for the consistent actor (mean_Trial 4_consistent_ = 10.21 s; *SD*_Trial 4_ = 7.36 s; mean_Trial 5_consistent_ = 10.40 s, *SD*_Trial 5_ = 7.03 s) versus the inconsistent actor (mean_Trial 4_inconsistent_ = 9.57 s; *SD*_Trial 4_ = 7.63 s; mean_Trial 5_inconsistent_ = 11.10 s, *SD*_Trial 5_ = 7.58 s) (*β* = 0.23, 95% CI of *β* [−0.11, 0.57], *b* = 0.22, *t* = 1.32, *p* = .184; see Figure 5). That is, infants did not appear to notice when the inconsistent actor changed its behavior. Indeed, although infants looked approximately 1 second longer to the inconsistent actor’s inconsistent versus its consistent event, this difference did not reach significance, nor was it significantly different from the same difference (the change in looking between the fourth and fifth trials) for the consistent actor.

#### Preferential Looking Phase.

There were three ways in which we examined whether infants preferred the actor who was on average more prosocial. First, we ran a preregistered mixed-effects model on the data in the preferential looking phase. In this model, looking time was the dependent variable, with fixed effects of actor type (the more prosocial versus the more antisocial Actor; within-subjects), condition (Mostly Prosocial Inconsistent Actor/Mostly Antisocial Inconsistent Actor; between-subjects), and their interaction. Subject ID was a random effect. We centered the fixed effects. Analyses revealed only a main effect of actor type: Infants looked longer to the more prosocial actor (mean = 12.01 s, *SD* = 5.63 s) than to the more antisocial actor (mean = 9.64 s, *SD* = 4.98 s) (*β* = 0.44, 95% CI of *β* [0.05, 0.83], *b* = 2.37, *t*(96) = 2.22, *p* = .028; see Figure 4B). The non-significant interaction between actor type and condition (*p* = .228) indicated that infants were no more likely to prefer the more prosocial actor versus the more antisocial actor when the inconsistent actor was either mostly prosocial or mostly antisocial.

Second, after running the mixed-effects model, we realized that it did not account for the fact that both actors were present on screen at once during the preferential looking phase and amended our preregistration to include additional analyses on the proportion of time that infants looked to the more prosocial actor as in Experiment 1 (Figure 4C). We found that this proportion did not differ by condition in a two-sample *t*-test (proportion_mostly antisocial inconsistent actor condition_ = 0.52, proportion_mostly prosocial inconsistent actor condition_ = 0.56, 95% CI [−0.14, 0.06], *t*(44) = −0.73, *p* = .468, Cohen’s *d* = 0.21). When collapsing across the two conditions, the proportion of time that infants looked at the more prosocial actor in Experiment 2’s preferential looking phase did not differ from 0.50 (chance) in a one-sample *t*-test (proportion = 0.54, 95% CI [0.49, 0.60], *t*(47) = 1.82, *p* = .074, Cohen’s *d* = 0.26). Although the finding was not significant, we note that the effect size suggests that there was a small effect.

Third, in exploratory analyses, we categorized infants as having looked more at either the more prosocial actor or the more antisocial actor, and we found that the number of infants who looked more at the prosocial actor versus the more antisocial actor did not differ by condition in a chi-squared test (*χ*^2^ = 1.35, *p* = .244). Of the 48 infants, 27 looked more at the more prosocial actor (binomial *p* = .470; Cohen’s *g* = 0.06).

### Discussion

In Experiment 2, we found mixed evidence that infants successfully evaluate inconsistent actors: When examining the raw data, the infants looked longer at the more prosocial actor in each condition. The proportion of looking at the more prosocial actor did not differ from chance in Experiment 2, but there was a small effect size suggestive that there may be a practical difference from 50%. However, we note that the evidence was overall considerably weaker in Experiment 2 than in Experiment 1, in which actors only behaved consistently. In addition to the null finding regarding the proportion of looking at the more prosocial actor in Experiment 2, there was a smaller raw looking time difference (with *β* = 1.00 in Experiment 1 and *β* = 0.44 in Experiment 2), and the number of infants looking more to the more prosocial actor did not differ from chance in Experiment 2.

We interpret the positive evidence with caution, given the overall mixed evidence. Still, analyses of the raw preferential looking time data are standard in the study of early social evaluation (e.g., Geraci et al., 2022; Hamlin & Wynn, 2011; Hamlin et al., 2010; Powell & Spelke, 2018; Woo, Liu, et al., 2024; Woo & Spelke, 2023a, 2023b). The analyses of the present raw preferential looking time data provide some positive evidence that infants may have evaluated inconsistent actors, contrasting with past work that has presented infants with inconsistent actors and found null findings (e.g., Shimizu et al., 2018; Steckler et al., 2017).

Why might infants have evaluated inconsistent actors (at least following the original preregistered analytic approach for Experiment 2), but not in past research? One possibility is that inconsistency is indeed difficult for infants to evaluate, and past work included additional inconsistencies that made the difficulty too great; these additional inconsistencies were reduced in the current studies. For instance, Steckler et al. (2017) used live puppet shows, which, given they are performed by human experimenters, inevitably vary somewhat from one trial to the next even when the nature of the unfulfilled goal scenario and the valence of the action remains consistent. In contrast, the current Experiment 2 utilized prerecorded videos of puppet shows that were entirely identical across trials, eliminating any extraneous inconsistencies. Likewise, Shimizu et al. (2018)’s inconsistent acts were from a novel scenario involving an entirely new unfulfilled goal relative to what infants had seen in habituation (retrieving a lost ball rather than opening a box); this inconsistency in scenario may have proven too significant for infants to evaluate actors who were also inconsistently prosocial/antisocial over time. In contrast, Experiment 2 presented infants with the same scenario throughout (always involving retrieving a lost ball); only the valence of the actor’s behavior (sometimes) changed. Together, the combination of Experiment 2 with past studies suggest that infants may be able to evaluate inconsistent actors if other forms of inconsistency are minimized.

Another possibility is that infants did not actually successfully engage in an averaging process to evaluate the inconsistent actors. Indeed, the evidence was mixed, and although infants’ attention increased somewhat in response to the inconsistent actor’s inconsistent behavior as in past work (e.g., Shimizu et al., 2018; see also, Taborda-Osorio et al., 2019; Tatone et al., 2015), the effect was not significant nor significantly different from their looking to the consistent actor’s behavior on the same trial. These null findings suggest that the present infants as a group may not have noticed the change in the inconsistent actor’s behavior. This would be striking, given past evidence in which infants have demonstrated sensitivity to changes in an actor’s usual behavior (e.g., actions directed at one object over another, as in Woodward, 1998; see also, Choi et al., 2018; Woo, Liu, & Spelke, 2024). Indeed, the use of video stimuli in our study means that the inconsistent actor’s *consistent* behavior was very consistent, relative to live stimuli in other research. We note that the lack of a significant finding may reflect that our study was underpowered to detect differences: Given our focus on social evaluation, our power analyses from pilot data were based on attention during the preferential looking trial rather than on event-based attention differences. We also note that other studies with similar sample sizes to ours have found evidence that infants are sensitive to changes in an actor’s usual behavior, but that these other studies have often involved habituation designs, whereby infants receive considerably more evidence of an actor’s usual behavior before the actor behaves differently (e.g., up to 14 trials, as in Woodward, 1998, in contrast to the four trials in our design). Thus, the lack of a significant finding here may reflect that we did not provide as much evidence as a habituation design involves.

Regardless of whether infants noticed the inconsistency, it is possible that initial consistency supports infants’ evaluations of inconsistent actors. In Experiment 2, the inconsistent actor always behaved in a way that was opposite that of the consistent actor, with the inconsistent actor switching its behavior during the last trial. Infants in the present experiment may have been able to evaluate the inconsistent actor based on its initial consistency. Evidence for this possibility comes from research on goal attribution. In classic research on goal attribution, Woodward (1998) found that after 6- and 9-month-old infants observe a hand grasping for one toy over another repeatedly, infants look longer when the hand later grasps the other, previously ignored toy, than when the hand later grasps the original object, as though infants had expected the hand to continue reaching for the same object. Building on Woodward’s findings, Duh and Wang (2019) examined 14-month-olds’ abilities to form such expectations from inconsistent behavior. They found that 14-month-olds can attribute goals to a person who behaves inconsistently, but only if the person’s initial behavior is consistent: 3 consecutive actions on the same object, preceding a switch in the target of the person’s actions. But when the person’s initial behavior was inconsistent (i.e., acting on two different objects in the first three trials), even when infants observed the same number of actions on an object, they did not attribute a goal to the person. It is possible that infants in the present work must also initially observe consistent behavior to evaluate inconsistent actors.

We designed Experiment 3 to attempt to conceptually replicate the findings of Experiment 2. A conceptual replication is important, given concerns about the timing of inconsistency and the mixed or weak evidence in Experiment 2 that infants were indeed evaluating inconsistent actors.

## EXPERIMENT 3: EVALUATIONS OF INCONSISTENT ACTORS; INCONSISTENCY FIRST

We designed Experiment 3 to conceptually replicate the preferential looking findings of Experiment 2 and address questions about how the timing of the inconsistency impacted results in Experiment 2. In Experiment 3, the inconsistent actor performed its unusual act first, before switching its behavior and acting in the opposite way 4 times. If the positive preferential looking findings from Experiment 2 resulted from infants ignoring the final inconsistent act and/or focusing entirely on what happened first, if infants require evidence of consistency over at least three trials before they can make sense of inconsistency (as in Duh & Wang, 2019), or if the positive preferential looking findings were not robust (given the mixed evidence in Experiment 2), then infants should not prefer the actor who is on average more helpful in Experiment 3. But if infants indeed evaluate inconsistent actors by averaging across all observed behaviors, regardless of the timing of inconsistency, then infants in Experiment 3 should still prefer the actor who is on average more helpful.

### Methods

#### Participants.

Sixty-nine 11-month-olds (42 girls; mean age = 10 months, 29 days; range = 10;15–11;20) participated. Infants were randomly assigned to the “Mostly Antisocial Inconsistent Actor” Condition (*n* = 35) or the “Mostly Prosocial Inconsistent Actor” Condition (*n* = 34).

##### Sample Size Justification.

We used previously collected data (from Experiment 2 and a pilot study) for power analyses in order to determine an appropriate sample size. In these data (*n* = 57), we examined the amount of time that infants looked at the more prosocial over the less prosocial actor by condition. In these data, infants preferred looking at the more prosocial actor.

We ran a power analysis to determine what the sample size should be to detect a looking preference of the same effect size with sufficient power in the mixed-effects model detailed above. With 64 infants (32 per condition), we would have 83% power to detect a looking preference for the more prosocial actor. More families responded to recruitment efforts than expected, leaving us with a total of 69 infants.

#### Procedures.

**Figure 6.**
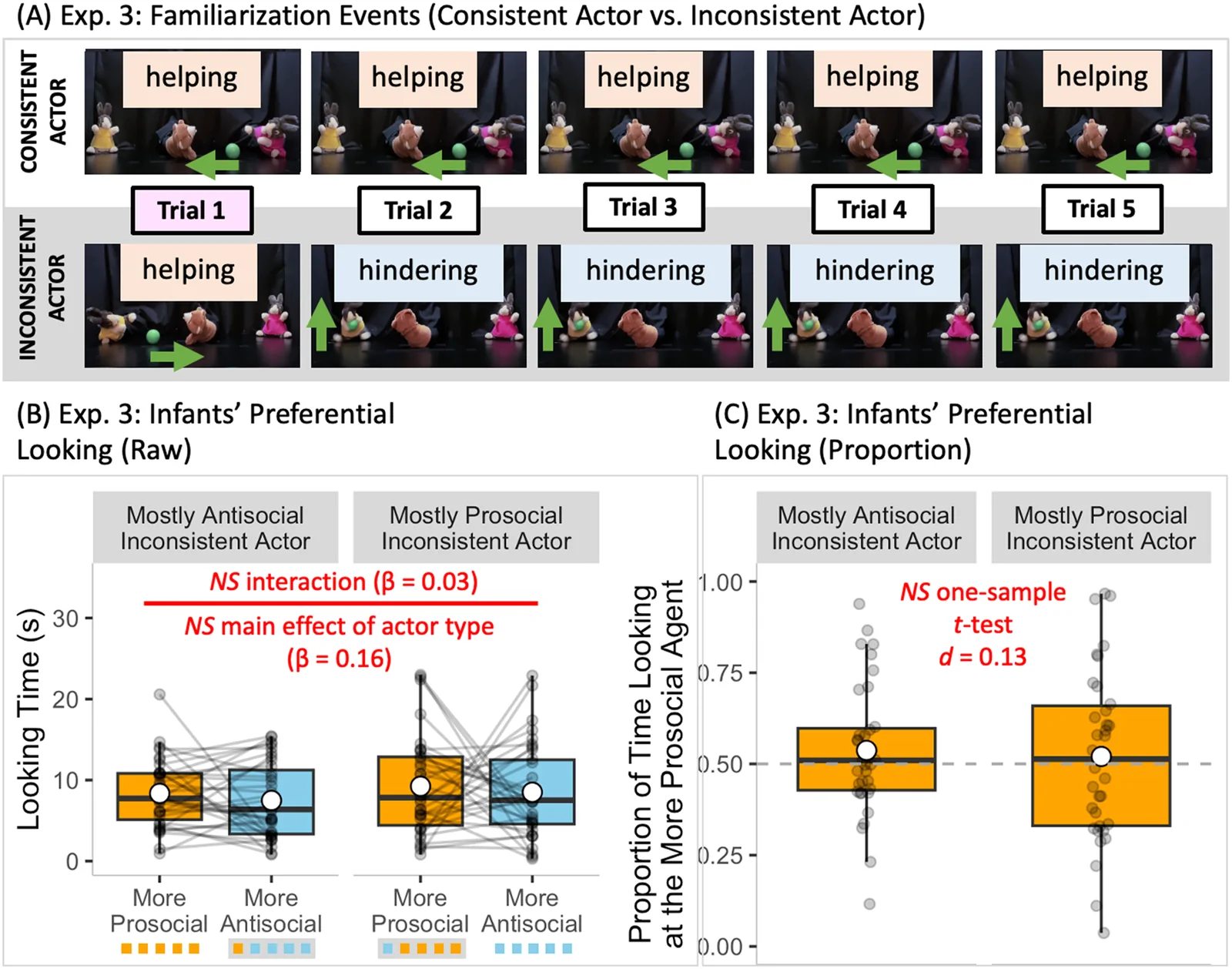
Experiment 3’s familiarization events (A) and infants’ preferential looking as raw data (B) and in terms of the proportion of time looking at the more prosocial actor (C). Orange indicates helping or prosocial behavior, blue indicates hindering or antisocial behavior, and purple indicates the trial for which the inconsistent actor’s behavior was different than it was for its other trials. The screenshots in panel A are representative of Experiment 3’s “Mostly Antisocial Inconsistent Actor” Condition; the “Mostly Prosocial Inconsistent Actor” Condition is not shown. Following the familiarization events, we assessed infants’ evaluations with a preferential looking phase (as in Experiment 1; see Figure 2B). In panel B, the colored boxes below box plots indicate the behaviors that an actor engaged in (helping in orange, hindering in blue) across its five trials; and the colored boxes in gray indicate that that actor was inconsistent. In the box plots, white circles indicate means, horizontal lines within boxes indicate medians, pairs of connected dots indicate data from a single child, and boxes indicate interquartile ranges. The beta coefficients (*β*) and Cohen’s *d* are measures of standardized effect size (*NS* *p* > .05; two-tailed).

The procedure was like that of Experiment 2, except that the inconsistent actor’s unusual act took place in its first trial (see Figure 6A).

### Results

#### Looking at Changes in Behavior.

Given that the change in behavior occurred between trials 1 and 2, before collecting data, we did not plan to examine expectations of consistency. In exploratory analyses, we nevertheless examined whether infants’ attention changed between trials 1 and 2, depending on whether the actor’s behavior had or had not changed. This model was like that of Experiment 1, except that the trials were 1 and 2, rather than 4 and 5.

We first fit both a gamma distribution and a normal distribution to the data. We found that a gamma distribution fit the data better than did a normal distribution. We therefore ran a gamma mixed-effects model. There was a significant main effect of trial order, such that infants looked less in the second trial (mean = 11.43 s, *SD* = 7.93) than in the first trial (mean = 13.59 s, *SD* = 8.02 s) for a given actor. However, the three-way interaction between actor type, trial order, and the usual act of the inconsistent actor was not significant (*p* = 0.391), nor was the two-way interaction between trial order and actor type (*p* = 0.152). That is, the effect of trial order did not differ depending on whether the actor was Inconsistent or Consistent, in either of the two conditions. We do not interpret these findings as evidence for or against infants’ expectations for consistency, given that infants only had one trial of evidence before the switch in behavior, and infants may not have formed strong expectations based on one trial.

#### Preferential Looking Phase.

As in Experiment 2, there were three ways in which we examined whether infants preferred the actor who was on average more prosocial. First, we ran a preregistered mixed-effects model on the data in the preferential looking phase using the same model as in Experiment 2. None of the main effects and interactions were significant (all *p*s > .41; Figure 6B). Infants did not differ in their looking to the more prosocial actor (mean = 8.79 s, *SD* = 5.20 s) and to the more antisocial actor (mean = 7.99 s, *SD* = 5.20 s) during the preferential looking trial.

Second, as in the previous two experiments, we realized that after running the mixed-effects model that it did not account for the fact that both actors were present on screen at once during the preferential looking phase and amended our preregistration. We found that the proportion of time that infants looked at the more prosocial actor in the preferential looking phase did not differ by condition in a two-sample *t*-test (proportion_mostly antisocial inconsistent actor_ = 0.53, proportion_mostly prosocial inconsistent actor_ = 0.52, 95% CI [−0.08, 0.11], *t*(61) = 0.32, *p* = .747, Cohen’s *d* = 0.07; Figure 6C). When collapsing across the conditions, the proportion of time that infants looked at the more prosocial actor in Experiment 2’s preferential looking phase did not differ from 0.50 (chance) in a one-sample *t*-test (proportion = 0.52, 95% CI [0.47, 0.57], *t*(68) = 1.15, *p* = .253, Cohen’s *d* = 0.13).

Third, in exploratory analyses, we categorized infants as having looked more at either the more prosocial actor or the more antisocial actor. Of the 69 infants, 35 looked more at the more prosocial actor (binomial *p* = 1.00; Cohen’s *g* = 0.00). The number of infants who looked more at the prosocial actor versus the more antisocial actor did not differ by condition in a chi-squared test (*χ*^2^ = 0.00, *p* = 1.00).

### Discussion

In both conditions of Experiment 3, for which the inconsistent actor’s unusual act occurred first, infants did not prefer the actor who had been on average more prosocial. These findings stand in contrast with the positive findings that infants looked longer at the more prosocial actors in Experiment 2, for which the inconsistent actor’s unusual act occurred on its last trial. Together, these findings suggest that infants may not evaluate inconsistent actors by averaging across observed behaviors.

## GENERAL DISCUSSION

In the present experiments, we first replicated the finding that infants prefer individuals who are consistently helpful (4×) over agents who are consistently unhelpful (4×) using an online testing platform with preferential looking as a dependent variable; evidence was positive across 3 distinct analyses (Experiment 1). In Experiment 2, we found mixed evidence that 11-month-old infants evaluate individuals who behave inconsistently last (1×) after several consistent behaviors (4×); our primary preregistered analysis showed positive evidence, but two additional analyses did not. Finally, in Experiment 3 we found no evidence that infants evaluate inconsistent actors when they act inconsistently first (1×) and then consistently (4×). Together, these findings suggest that although infants readily distinguish between consistently prosocial and antisocial actors, they do not reliably evaluate actors that have behaved inconsistently.

### Capacities to Evaluate Inconsistent Actors in the First Year

Experiment 2 provided some positive evidence that 11-month-old infants can evaluate inconsistent actors, using our preregistered analysis on raw looking times. This positive evidence stands in contrast with past research that has found that infants in the first year struggle to evaluate actors who behave inconsistently (Shimizu et al., 2018; Steckler et al., 2017), suggesting that one or more of the adjustments we made to improve performance relative to past work may have helped infants to evaluate inconsistent actors; here, we tested older infants, we used a different helper/hinderer paradigm and a different preference measure, and we provided infants more evidence of the consistent act. However, infants’ success was weak and mixed in Experiment 2, in that it did not stand up to multiple ways of testing the same hypothesis. Further, here and in past work (Shimizu et al., 2018; Steckler et al., 2017), even successful infants might have succeeded by simply ignoring an inconsistent actor altogether, and simply approaching consistently prosocial actors (here, in the “Mostly Antisocial Inconsistent Actor” condition) and avoiding consistently antisocial actors (here, in the “Mostly Prosocial Inconsistent Actor” one). Indeed, in Experiment 2, infants as a group did not reliably notice the inconsistent actor’s change in behavior, suggesting that their engagement with the inconsistency was relatively minimal. Finally, infants’ limited success at evaluating inconsistent actors was only observed when the inconsistent act happened last (as in Experiment 2); infants failed when the inconsistent act happened first (and the two actors initially behaved the same way; as in Experiment 3). Overall, we take these findings as evidence that, overall, 11-month-old infants’ ability to evaluate inconsistent actors is seriously limited, if present at all.

Adults’ evaluations can be guided by negativity biases and primacy biases. We found no evidence that a negativity bias impacted infants’ evaluations, in either Experiments 2 or 3: The patterns of findings of both expectations and preferential looking did not differ depending on whether the inconsistent actor was mostly prosocial or mostly antisocial. Thus, although adults often weigh negative information more heavily than they do positive information, and although some past work suggests that infants do too (e.g., Chae & Song, 2018; Hamlin & Baron, 2014; Hamlin & Wynn, 2012; Hamlin et al., 2010), infants in the current experiments did not.

In contrast, results across Experiments 2 and 3 provide some, quite tentative, evidence that infants are subject to a primacy bias, in which actors’ initial behaviors are prioritized in social evaluation: Infants did better in Experiment 2 when the inconsistent action came last than in Experiment 3 when it came first. This possibility may explain the findings of Shimizu et al. (2018); although Shimizu et al. found that 15- to 18-month-old toddlers (but not younger infants) evaluated inconsistent actors, Shimizu et al. only ever showed participants stimuli in which the inconsistent and consistent actors started out behaving differently. Thus, toddlers may have evaluated the actors based on these initially distinct behaviors. That said, given the mixed findings in Experiment 2; further studies are clearly necessary to examine whether a primacy bias guides infants’ social evaluations. Further, if such a primacy bias indeed exists in development it is unclear what it consists of: It could be that infants can only handle inconsistencies after an actor first behaves consistently for some time, supporting a stable first impression that facilitates weighting that information relatively heavily.

Alternatively, infants may have difficulty processing any inconsistency at all, and so they may ignore it, focusing on the inconsistent actor’s earlier behavior and/or instead simply responding to the consistent actor’s behavior. Indeed, in Experiment 2 infants did not look significantly longer when the inconsistent actor’s behavior changed. That said, the data trended in that direction, and it is unclear if the experiment was simply underpowered to detect a reliable looking time effect. Future research should tease apart these possibilities by varying when in a series of actions an actor’s inconsistent action occurs, beyond what was manipulated in the present experiments (inconsistency in the first versus the last act).

Why is inconsistency so difficult for infants? One possibility is that infants may just not be very good at social evaluation, and so any inconsistency disrupts their present, but weak, ability to evaluate prosocial versus antisocial actors. Indeed, although we successfully replicated infants’ preference for consistent prosocial versus antisocial actors in the current studies, there are numerous failed replications of infant social evaluation effects in the literature (Cowell & Decety, 2015; Lucca et al., 2025; Nighbor et al., 2017; Salvadori et al., 2015; Schlingloff et al., 2020); these failures may indicate that early capacities for social evaluation are simply fragile. If infants’ evaluative capacities are indeed fragile, then behavioral inconsistency may prove challenging in that it overwhelms an already weak system. This could explain the weaker, mixed evidence that we observed in Experiment 2, and the fully null findings that we observed in Experiment 3.

On the other hand, numerous studies have shown that infants’ and toddlers’ evaluations of consistent actors can be complex, incorporating information about actors’ intentions, knowledge, and beliefs (Geraci et al., 2022; Geraci & Surian, 2023; Hamlin, 2013a; Kanakogi et al., 2017; Woo & Spelke, 2023b; Woo et al., 2017); their past behaviors (Hamlin, 2014; Hamlin et al., 2011; Jin et al., 2024; Singh, 2020; Ting et al., 2019); and their relationships to infants themselves (Burns & Sommerville, 2014; Hamlin, Mahajan, et al., 2013). Interestingly, infants and toddlers appear not to be overwhelmed or overtaxed in these studies, wherein the constructs being tested are quite complex (for instance, requiring keeping track of the distinct behaviors of many different actors at once) but in which no individual actor ever behaves inconsistently. This could indicate that inconsistent acts performed by the same actor are *particularly* taxing for infants’ social evaluative systems. Future studies could examine whether contextual explanations for within-agent inconsistency (for instance, an agent whose actions vary across time but only when interacting with new targets) could enable infants to succeed at evaluating inconsistent actors.

The present findings add to a growing body of research on the development of children’s ability to make sense of unexplained behavioral inconsistency (e.g., Shimizu et al., 2018; Steckler et al., 2017). In a recent study, Yucel et al. (2025) found that 4-, 6-, and 8-year-old children preferred consistently prosocial actors over consistently antisocial actors, but when an actor behaved inconsistently (sometimes prosocial, sometimes antisocial), only the older children reliably distinguished inconsistent actors from consistently prosocial and from consistently antisocial actors; the 4-year-old children did not distinguish the inconsistent actors from the consistent ones. These findings in 4-year-old children are consistent with the present findings in infants. It may be that younger children and infants require explanations (e.g., that prosocial and antisocial actions are directed towards different agents) to make sense of inconsistency.

### Capacities to Evaluate Helpers and Hinderers in the First Year

Beyond providing evidence that infants experience difficulty evaluating inconsistent actors, the present research speaks to infants’ developing capacities to engage in social evaluation. As reviewed above, there have been several failed replications of the finding that infants prefer helpers over hinderers (Cowell & Decety, 2015; Lucca et al., 2025; Nighbor et al., 2017; Salvadori et al., 2015; Schlingloff et al., 2020). The present paper successfully replicates that finding in 11-month-old infants (Experiment 1) and provides some evidence (albeit mixed) that 11-month-old infants can evaluate inconsistent actors when their inconsistent actors come last (Experiment 2).

There are multiple possible explanations for why we found positive evidence in Experiment 1, where other research has failed to replicate the finding that infants prefer helpers over hinderers. First, as Margoni and Surian (2018) noted, the effect size for the ball scenario that we adapted may be larger than that of other helping scenarios such as the hill and box scenarios, and those have been the focus of most replication work to date. Second, we used a different dependent variable: preferential looking, rather than reaching. It is possible that, looking may be a more sensitive or less noisy measure than is reaching (for discussion, see Woo et al., 2025). Amongst other differences, reaching involves a one-shot, manual forced-choice, providing very little room for error or variation. In contrast, looking involves attention that is measured continuously over a relatively long period. Moreover, reaching measures have exclusively been utilized during live interactions with an experimenter who is often novel, as they must remain unaware of conditions. By contrast, preferential looking can be measured with no experimenter involvement at all (and indeed, over the internet from infants’ familiar home environments). Thus, reaching tasks may be more dependent on the level rapport established between infant and experimenter (see, e.g., Cortes Barragan & Dweck, 2014 for related arguments with infant helping rates) and infants’ overall level of comfort than are looking tasks; we note that the recent and large-scale failure of infants to prefer helpers in the hill paradigm (Lucca et al., 2025) was conducted very shortly following COVID-19 lockdowns that included significant reductions in social contact (Hale et al., 2021), and infants may thus have felt particularly wary of novel experimenters during this period. We look forward to future research that explores these possibilities.

### Conclusion

Although we found some positive evidence in one experiment that 11-month-old infants evaluated inconsistent actors, the overall evidence was mixed, and we did not conceptually replicate that evidence in a further experiment. Moreover, infants did not appear to notice inconsistency in behavior in any experiment. The present experiments contribute to a growing body of research that infants can experience difficulty evaluating inconsistent actors, even when the inconsistency is very low (1 of 5 acts) and even when a separate group of infants succeeded in evaluating actors who consistently performed the very same acts.

## ACKNOWLEDGMENTS

We thank the families who participated in these studies; Julian Kwong, Gia Kakkar, Angela Zhu, Anni Persson, Brittany Tsang, Mehek Budshah, Fibha Khan, Caroline Mawhinney, and Chloe Fichter for research assistance; Francis Yuen for helpful discussion; and ChildrenHelpingScience.com for support with recruitment.

## FUNDING INFORMATION

There is no funding to report.

## AUTHOR CONTRIBUTIONS

B.M.W.: Conceptualization; Formal analysis; Investigation; Methodology; Project administration; Visualization; Writing – original draft; Writing – review & editing. A.T.: Investigation; Visualization. J.K.H.: Conceptualization; Formal analysis; Methodology; Project administration; Resources; Supervision; Writing – original draft; Writing – review & editing.

## DATA AVAILABILITY STATEMENT

The stimuli, data, and code are hosted at https://osf.io/879ys/.
